# Experimental Infection of Cats and Dogs with West Nile Virus

**DOI:** 10.3201/eid1001.020616

**Published:** 2004-01

**Authors:** Laura E. Austgen, Richard A. Bowen, Michel L. Bunning, Brent S. Davis, Carl J. Mitchell, Gwong-Jen J. Chang

**Affiliations:** * Colorado State University, Fort Collins, Colorado, USA; † Centers for Disease Control and Prevention, Fort Collins, Colorado, USA

**Keywords:** West Nile virus, flavivirus, dog, cat

## Abstract

Domestic dogs and cats were infected by mosquito bite and evaluated as hosts for West Nile virus (WNV). Viremia of low magnitude and short duration developed in four dogs but they did not display signs of disease. Four cats became viremic, with peak titers ranging from 10^3.0^ to 10^4.0^ PFU/mL. Three of the cats showed mild, non-neurologic signs of disease. WNV was not isolated from saliva of either dogs or cats during the period of viremia. An additional group of four cats were exposed to WNV orally, through ingestion of infected mice. Two cats consumed an infected mouse on three consecutive days, and two cats ate a single infected mouse. Viremia developed in all of these cats with a magnitude and duration similar to that seen in cats infected by mosquito bite, but none of the four showed clinical signs. These results suggest that dogs and cats are readily infected by WNV. The high efficiency of oral transmission observed with cats suggests that infected prey animals may serve as an important source of infection to carnivores. Neither species is likely to function as an epidemiologically important amplifying host, although the peak viremia observed in cats may be high enough to infect mosquitoes at low efficiency.

 The appearance of the West Nile virus (WNV) in New York in 1999 and the subsequent establishment and spread of the virus have aroused public anxiety about the potential of companion animals to become infected, show illness and die, and transmit WNV to other species, including humans. Little information is available concerning the susceptibility of dogs and cats to WNV infection. Approximately one third of a large sample of dogs from a WNV-endemic region of South Africa had neutralizing antibody to WNV, and viremia was detected in one of three dogs inoculated with a large dose of WNV (*1*). Recently, WNV was isolated from the brain of a cat with “neurologic disease” (*2*), but nothing is known about the natural history of WNV infection in this species. Most dogs and cats spend at least some time out of doors and thus risk exposure from hematophagous insect vectors. Also, as carnivores, they may be exposed to WNV through ingestion of infected small mammals and birds, which are known to have large quantities of WNV in blood and tissue during the course of infection (*2*,*3*). Since there are an estimated 68 million companion dogs and 73 million pet cats in the United States alone (*4*), evaluating their response to WNV infection, assessing their potential to serve as amplifying hosts for this virus, and obtaining some estimate of the clinical consequences of infection are important concerns.

## Materials and Methods

### Animals and Examinations

 Four young adult female, crossbred hounds (Harlan, Indianapolis, IN) and eight adult, female domestic, crossbred cats (Liberty Laboratories, Waverly, NY) were used. All were purchased as specific pathogen-free animals. Each cat was negative for antibodies to feline immunodeficiency virus and negative for feline leukemia virus antigen in serum. Animals were gang housed by species under animal biosafety level three conditions with ad libitum access to food and water. They were examined clinically by a veterinarian twice daily for the duration of the study but did not receive formal neurologic evaluations. Body temperature and general appearance were recorded twice daily from days –0.5 to 7 or 9 (day 0 being the day of infection). With few exceptions, blood was collected twice daily from days –1 (dogs) or 0 (cats) to 9, at 2 and 3 weeks postinfection, and at the time of euthanasia. For the animals exposed to WNV through mosquito bites, blood was collected into EDTA tubes for hematology once daily from days –1 (dogs) or 0 (cats) to 10, and on days 14 and 21. Hematologic parameters (erythrocyte and leukocyte counts, platelet counts, plus relative and absolute mononuclear and granulocyte counts) were evaluated by using a QBC-V analyzer (Clay-Adams, Parsippany, NJ). Saliva was collected from mosquito-inoculated animals once daily from days 0 to 7 by swabbing the oral cavity with a cotton swab premoistened with BA-1 medium (M-199 salts, 1% bovine serum albumin, 250 mg/L sodium bicarbonate, 100 U penicillin G/mL, 100 μg/mL streptomycin, 1 μg/mL amphotericin B in 50 mM Tris, pH 7.6). All animals were euthanized between days 22 and 50 after infection by pentobarbital overdose and necropsied; their carcasses were incinerated within the containment facility.

 Hematologic and body temperature data were analyzed by repeated measures analysis of variance and compared to a baseline by using the differences of least squares means statistical test (Proc Mixed, SAS Statistical Software, SAS Institute, Cary, NC). Baseline values were obtained on day 0 (body temperature) or represent the mean of values obtained on days 0 and 21 (hematologic data).

### Infection by Mosquito Feeding

Four dogs and four cats were exposed to WNV through the bites of infective *Aedes albopictus* mosquitoes. The mosquitoes were from a colony strain from Lake Charles, Louisiana, which had been maintained in the insectary for several years with periodic additions of field-collected stock. Mosquitoes were reared in an insectary maintained at 26.7°C, approximately 80% relative humidity and a light:dark cycle of 16:8 hours. Larvae were fed liver powder and rabbit chow ad libitum. Cohorts of adult female *Ae. albopictus* 3–5 days of age were inoculated intracthoracically with approximately 170 Vero PFU of the NY99 strain of WNV, isolated originally from an infected crow. Inoculated mosquitoes were placed in 3.8-L cages, given 5% sucrose for maintenance, and incubated for 12 days under the same insectary conditions described as before virus inoculation. On the afternoon before feeding on dogs and cats, mosquitoes were transferred to cylindrical cartons with netting on both ends, denied sucrose solution, and held overnight with a pad moistened lightly with water on their cages.

 Cats were lightly anesthetized with ketamine, and dogs were sedated with xylazine during mosquito feeding. Before feeding, a 10- to 15-cm diameter area of fur over the thorax was clipped using a #40 blade. Feeding was accomplished by holding the mosquito cage against the clipped area of skin for approximately 5 minutes. Individual lots of 14 mosquitoes were given the opportunity to feed on each animal, then transported to a secure laboratory. They were anesthetized with CO_2_ and sorted on wet ice. Three engorged mosquitoes per animal were ground individually in BA-1 using a mixer mill. Samples were then clarified in a refrigerated microcentrifuge (5,000 x *g* for 5 min) and tested for virus by plaque assay.

### Oral Exposure to WNV

Four cats were exposed to WNV by ingestion of mice previously infected by intraperitoneal inoculation of approximately 100 PFU of WNV (NY99), incubated for 5 to 8 days, and euthanized by CO_2_ inhalation immediately before being given to the cats. To estimate the minimal virus load of the mice, cohorts of the mice fed to cats were euthanized at the same time and frozen at –70°C; later, their brains were assayed for virus by plaque assay. The cats were housed individually for feeding, by leaving the infected mouse on a plate overnight. In most but not all cases, the mouse was observed to have been eaten within 1 hour. If a cat had not eaten the mouse by the following morning, it was not considered an exposure, the mouse was discarded, and another mouse was presented the following evening. Two cats were fed one infected mouse each on three successive days, and two other cats were each fed a single infected mouse.

### Plaque and Neutralization Assays

Serum, saliva, mosquitoes, and mouse brain homogenates were tested for virus concentration by plaque assay. Briefly, serial 10-fold dilutions of the samples were made in BA-1 medium and 0.1-mL volumes were inoculated onto monolayers of Vero cells in 6-well tissue culture plates. The plates were incubated at 37°C in an atmosphere containing 5% CO_2_, then overlaid with 3 mL of 0.5% agarose in M-199 medium supplemented with 5% fetal bovine serum and antibiotics. After 48 hours, a second 3-mL overlay containing 0.004% neutral red was added. Plaques were scored on days 3 and 4 of incubation. Selected serum samples were tested for antibodies to WNV with a 90% plaque-reduction neutralization test, as described previously (*5*).

## Results

The number of mosquitoes that fed on dogs 1 to 4 and cats 1 to 4 ranged from 8 to 11. The quantity of WNV present in the three mosquitoes assayed from each animal after feeding ranged from 10^7.4^ to 10^7.9^ PFU per mosquito.

Viremia was detected in the four dogs from 12 to 60 hours after exposure to WNV (Table). The quantity of virus in serum was low and fluctuated, and viremia was not detected past day 4.5. The peak viremia detected in the dogs ranged from 10^1.6^ to 10^2.2^ PFU/mL. Virus was not isolated from any of the oral swab samples. Clinical signs of disease were not observed in any of the infected dogs. They ate readily and remained energetic and afebrile, with the exception of dog 4, which had a slightly elevated body temperature 12 hours after mosquito feeding (Figure). Dog 4 was in proestrus and estrus during the challenge period. Statistical analysis of canine hematologic changes indicated a decrease in leukocyte count from days 2 through 7 and in hematocrit from days 4 through 9. However, the least squares means for those parameters were within the normal clinical reference range throughout the sampling period, suggesting that hematologic analysis is not clinically predictive. At necropsy on day 50, gross abnormalities were not observed in any dog.

**Table:** West Nile virus titers in serum of dogs and cats following bites of virus-infected mosquitoes or ingestion of virus-infected mice

Route of Exposure	Animal	WNV virus titer (log_10_ PFU/mL serum) at days postexposure																		
		0	0.5	1.0	1.5	2.0	2.5	3.0	3.5	4.0	4.5	5.0	5.5	6.0	6.5	7.0	7.5	8.0	8.5	9.0
Mosquito	Dog 1	<1	1.6	<1	<1	<1	1	<1	<1	<1	<1	<1	<1	<1	<1	<1	-^a^	-	-	-
Mosquito	Dog 2	<1	<1	2	2	<1	2.2	2	<1	<1	<1	<1	<1	<1	<1	<1	-	-	-	-
Mosquito	Dog 3	<1	<1	<1	<1	<1	1	1.6	1.6	1	1	<1	<1	<1	<1	<1	-	-	-	-
Mosquito	Dog 4	<1	<1	1.6	1.6	<1	1.6	1.6	1.6	1	1	<1	<1	<1	<1	<1	-	-	-	-
Mosquito	Cat 1	<1	1.6	2.3	2.9	2.4	2.8	2.7	3.2	2	2	<1	<1	<1	<1	<1	-	-	-	-
Mosquito	Cat 2	<1	<1	2.1	2.3	2.4	2.7	2.8	3.0	2.6	1	<1	<1	<1	<1	<1	-	-	-	-
Mosquito	Cat 3	<1	<1	<1	1	1.6	1.9	2.0	2.8	3.4	3.0	<1	<1	<1	<1	<1	-	-	-	-
Mosquito	Cat 4	<1	<1	1	2.5	3.2	3.2	4.0	3.2	3.0	1	<1	<1	<1	<1	<1	-	-	-	-
Oral	Cat 14	<1	-	1	3.2	2.9	3.2	3.4	3.7	3.6	3.2	<1	<1	<1	<1	<1	-	-	-	-
Oral	Cat 16	<1	-	2	2.7	2.8	3.4	3.6	3.9	3.6	2.7	<1	1	<1	<1	<1	-	-	-	-
Oral	Cat 17	-	<1	<1	<1	<1	<1	<1	<1	1.8	1.0	1.6	2.1	2.5	2.3	1.8	1.0	1.3	<1	<1
Oral	Cat 18	<1	<1	<1	<1	<1	1.8	1.3	1.5	1.0	1.6	2.2	1.5	1.3	<1	<1	<1	<1	<1	<1

^a^-, Not done.

**Figure:**
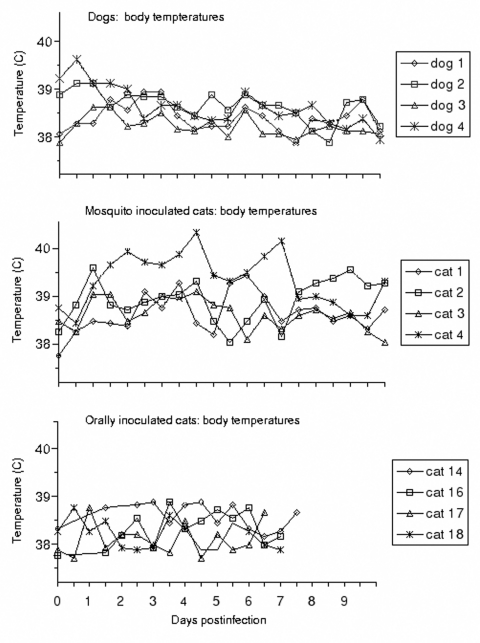
Body temperatures during the course of experimental West Nile virus infection. Reference ranges for clinically normal dogs and cats were considered to be 38.3°C–39.5°C and 38.0°C–39.2°C, respectively.

Viremia developed in the four cats exposed to WNV by feeding of infected mosquitoes 12 to 36 hours after exposure (Table). The peak titers of virus in serum ranged from 10^3.0^ to 10^4.0^ PFU/mL, and virus was not isolated from any samples collected >4.5 days after mosquito feeding. Three of the four cats (numbers 1, 2, and 4) displayed both lethargy and a fluctuant febrile response (Figure), but specific neurologic signs were not observed in any cat. Significant (p < 0.05) elevations of rectal temperature occurred from days 1 through 6. A number of isolated significant deviations from baseline in hematologic parameters were identified, but meaningful trends were not observed. Cat 4 was anemic at the time of challenge (hematocrit 21%), became more severely anemic during the first week after challenge (hematocrit as low as 13%), then rebounded by days 9 to 14 (hematocrit 18%-22%). WNV was not isolated from any of the oral swab samples. At necropsy on day 50, gross abnormalities were seen in cat 2 (pale, reticulated liver) and cat 4 (hydrocephalus and a nodular, fibrous spleen). These findings were considered incidental and unrelated to WNV infection; hydrocephalus in the absence of overt neurologic signs is not uncommon in cats (D. Baker, pers. commun.).

Clinical signs, including pyrexia and neurologic abnormalities, were absent throughout the study period in cats infected with WNV by ingesting three (n = 2) or one (n = 2) infected mice. Viremia developed in cats that ingested three mice (cats 14 and 16) within 24 hours of ingesting the first mouse (these cats were not bled at 12 hours), and viremia resolved by day 6 (Table). The peak viremia in these two animals was 10^3.7^ and 10^3.9^ PFU/mL, respectively. Cohort mice of those fed at the first of the three feedings yielded 10^9.5^ and 10^9.7^ PFU/g of brain. Cats 17 and 18 ingested a single mouse each; these two mice were euthanized approximately 2 days earlier in the course of their infection than those fed to cats 14 and 16, and virus titration of their cohorts showed 10^5.3^ and 10^6.5^ PFU/g of brain. Cats fed a single mouse had viremia of similar duration but delayed onset (day 4 and 2.5 for cats 17 and 18, respectively), and lower peak titer (10^2.5^ and 10^2.2^ PFU/mL serum) than occurred in the cats ingesting mice with higher virus content (Table). Neutralizing antibody titers to WNV of at least 1:10 developed in three of the four cats fed infected mice by day 14 postexposure (range 1:10-1:40, 90% neutralization). Cat 18, which had a neutralizing titer <1:10 on day 14, had a titer of 1:40 on day 21.

## Discussion

Little attention has been paid to dogs and cats as hosts for WNV, and neither species is a recognized natural host for this virus. However, considering the large populations of these animals in North America and their close association with humans, understanding their clinical response to infection and the likelihood that they might serve as amplifying hosts are important. Dog and cat owners have also expressed concern about the potential effects of WNV infection on their pets. Roughly one in three dogs from a WNV-endemic region of South Africa were found to have neutralizing antibody to WNV (*1*). More recently, 10 of 139 dogs and none of 10 cats sampled from New York City during the fall of 1999 were reported to have WNV-neutralizing antibody (*6*); the number of these animals, particularly the cats, that spent a considerable amount of time out of doors was not known. WNV was isolated from the brain of a cat from New York that displayed neurologic disease (*2*).

*Ae. albopictus* mosquitoes were used to challenge the animals in these experiments because they are known to be capable of transmitting WNV by bite (*7*,*8*) and to reproduce a natural route of infection. In addition, WNV has been isolated from *Ae. albopictus* in New York (*9*).

None of the four dogs infected by mosquito bite showed clinical signs of disease, and although each became viremic, the quantity of virus in blood was low and fluctuated considerably. These results are similar to those described by Blackburn and co-workers, who found no clinical signs and viremia in one of three dogs inoculated by subcutaneous and intravenous inoculation with a South African strain of WNV (*1*). Collectively, these observations and the paucity of clinical reports of WNV disease in dogs suggest that WNV infection in dogs is typically subclinical in nature.

Three of the four cats infected by mosquito bite showed mild, nonspecific signs of disease at times during the first week after challenge. Signs included lethargy and modest decreases in appetite, none to the extent that would typically alarm pet owners. Two of the four cats (numbers 2 and 4) did show periods of distinctly elevated body temperature. Cat 4 had a preexisting anemia, which may have contributed to the course of disease. The duration of viremia in these four animals ranged from 3.5 to 4.5 days, with peak titers between 10^3.2^ and 10^4.0^ PFU/mL.

 The oral transmission experiments conducted with cats were designed to determine whether carnivores are likely to become infected with WNV through eating infected birds or small mammals, which often contain high quantities of virus. Transmission of a vaccine strain of Japanese encephalitis virus to mice by instillation of virus into the oral cavity has previously been reported (*10*). Efficient transmission to adult mice by oral instillation of WNV has been reported (*3*), but similar attempts failed to transmit the virus (*11*). Further, isolation of WNV from a Red-tailed Hawk found dead in New York during midwinter was suggested to be due to predation, although the source of an infected prey animal at that time of year was not clear (*12*). Finally, milk-borne transmission from sheep to humans of the tick-borne encephalitis flavivirus has been reported (*13*). The current report provides the first experimental demonstration of oral transmission of a flavivirus using methods that approximate a natural type of exposure in mammals. The four cats that ate one or three infected mice became infected, indicating that this route of transmission is quite efficient. Whether such transmission results simply from oral exposure to virus, depends upon inoculation through the oral mucosa by small splinters of bone, or proceeds through some other pathway is not known. Nonetheless, transmission of WNV by predation or scavenging seems another likely important route of WNV transmission in mammals and birds.

The magnitude of WNV viremia necessary to efficiently infect feeding mosquitoes varies with mosquito species. Based on experiments using viremic chicks as a blood source, Jupp estimated 10% infection thresholds for South African strains of *Culex univittatus*, *Cx. pipiens*, *Cx. quinquefasciatus*, and *Cx. theileri* of <10^2.7^, 10^2.7^, 10^2.7^ and <10^4.1^ adult-mouse lethal dose (LD)_50_/mL, respectively (*14*). More recently, Turell and co-workers fed a variety of mosquito species from the eastern United States on viremic chicks (*15*). Infection rates in these experiments ranged from 0% to 17% for mosquitoes ingesting blood with a WNV titer of 10^5.2^ Vero cell PFU/mL, and from 0% to 92% for mosquitoes that fed on chicks circulating 10^7.0^ (+ 0.3) PFU/mL, respectively. We did not test the ability of any species of mosquito to become infected by feeding on viremic dogs or cats. The low serum virus titers reported here and by Blackburn and colleagues (*1*) suggest that dogs are very unlikely to serve as an amplifying host for WNV. In contrast, the peak viremias observed in all of the mosquito-fed and orally exposed cats suggest that they may support infection of mosquitoes, albeit with low efficiency relative to many avian hosts.
